# A High-Temperature Transient Hot-Wire Thermal Conductivity Apparatus for Fluids

**DOI:** 10.6028/jres.096.014

**Published:** 1991

**Authors:** R. A. Perkins, H. M. Roder, C. A. Nieto de Castro

**Affiliations:** National Institute of Standards and Technology, Boulder, CO 80303; Departamento de Quimica, Universidade de Lisboa, R. Ernesto Vasconcelos, Bloco Cl, 1700 Lisboa, Portugal

**Keywords:** argon, heat capacity, nitrogen, radiation correction, thermal conductivity, thermal diffusivity, toluene, transient hot-wire

## Abstract

A new apparatus for measuring both the thermal conductivity and thermal diffusivity of fluids at temperatures from 220 to 775 K at pressures to 70 MPa is described. The instrument is based on the step-power-forced transient hot-wire technique. Two hot wires are arranged in different arms of a Wheatstone bridge such that the response of the shorter compensating wire is subtracted from the response of the primary wire. Both hot wires are 12.7 µm diameter platinum wire and are simultaneously used as electrical heat sources and as resistance thermometers. A microcomputer controls bridge nulling, applies the power pulse, monitors the bridge response, and stores the results. Performance of the instrument was verified with measurements on liquid toluene as well as argon and nitrogen gas. In particular, new data for the thermal conductivity of liquid toluene near the saturation line, between 298 and 550 K, are presented. These new data can be used to illustrate the importance of radiative heat transfer in transient hot-wire measurements. Thermal conductivity data for liquid toluene, which are corrected for radiation, are reported. The precision of the thermal conductivity data is ± 0.3% and the accuracy is about ±1%. The accuracy of the thermal diffusivity data is about ± 5%. From the measured thermal conductivity and thermal diffusivity, we can calculate the specific heat, *C_p_*, of the fluid, provided that the density is measured, or available through an equation of state.

## 1. Introduction

The transient hot-wire method is widely accepted as the most accurate technique for fluid thermal conductivity measurements at physical states removed from the critical region proper [1]. The method is very fast relative to steady state techniques. The duration of a typical experiment is about 1 s when 250 temperature rises are measured. Normally the experiment is completed before free convection can develop in the fluid. If free convection is present, it is easy to detect because it results in a pronounced curvature in the graph of temperature rise versus the logarithm of time.

In addition to the thermal conductivity, thermal diffusivity can be measured with transient hot-wire instruments. With an appropriate design of the instrument [2], measurements of fluid thermal diffusivity can be made with reasonable accuracy over wide ranges of density. The heat capacity of a fluid can then be obtained from the measurements of thermal conductivity and thermal diffusivity, provided that the density is known or available from an equation of state.

## 2. Method

The transient hot-wire system is considered to be an absolute primary instrument [1]. The ideal working equation is based on the transient solution of Fourier’s law for an infinite linear heat source [3]. The temperature rise of the fluid at the surface of the wire, where *r =r*_0_, at time *t* is given by

$$\begin{array}{l} \Delta T_{\text{ideal}}\left(r_{0},t\right)=\frac{q}{4\pi \lambda }\ln\left(\frac{4at}{r_{0}^{2}C}\right)=\frac{q}{4\pi \lambda }\ln\left(\frac{4a}{r_{0}^{2}C}\right)\\ +\frac{q}{4\pi \lambda }\ln\left(t\right). \end{array}$$ (1)

In eq (1)*q* is the power input per unit length of wire, λ is the thermal conductivity, *a* = λ/ρ*C*_p_ is the thermal diffusivity of the fluid, ρ is the density, *C_p_* is the isobaric heat capacity, and *C* = e^γ^ = 1.781… is the exponential of Euler’s constant. We use eq (1) and deduce the thermal conductivity from the slope of a line fit to the Δ*T*_ideal_ versus ln(*t*) data. The working equation for the thermal diffusivity is

$$a=\frac{r_{0}^{2}C}{4t\prime }\exp\left[\frac{4\pi \lambda \Delta T_{\text{ideal}}\left(r_{0\prime }t\prime \right)}{q}\right].$$ (2)

The thermal diffusivity is obtained from λ and a value of Δ*T*_ideal_, from the fit line, at an arbitrary time *t*′. We normally select *t′* to be 1 s in our data analysis, as discussed in reference [2].

The thermal conductivity is reported at the reference temperature *T*, and density ρ_r_ defined in eq (3) below. The thermal diffusivity calculated from eq (2) must be referred to zero time, that is, the equilibrium or cell temperature. In summary, the thermal conductivity and the thermal diffusivity evaluated by the data reduction program are related to the reference state variables and to the zero time cell variables as follows:

$$\begin{array}{l} \lambda =\lambda \left(T_{r},\rho_{r}\right),\\ T_{r}=T_{0}+0.5\left(\Delta T_{\text{initial}}+\Delta T_{\text{final}}\right),\\ \rho_{r}=\rho \left(T_{r},P_{0}\right),\\ a=a\left(\rho_{0}T_{0}\right)=\frac{\lambda \left(T_{0},\rho_{0}\right)}{\rho_{0}{\left(C_{p}\right)}_{0}},\\ \rho_{0}=\rho \left(T_{0},P_{0}\right),\;\text{and}\\ {\left(C_{p}\right)}_{0}=C_{p}\left(T_{0},P_{0}\right), \end{array}$$ (3)

where *T*_0_ is the equilibrium temperature and *P*_0_ is the equilibrium pressure at time *t* =0.

The experimental apparatus is designed to approximate the ideal model as closely as possible. There are, however, a number of corrections which account for deviations between the ideal line-source heat transfer model and the actual experimental heat transfer situation. The ideal temperature rise is obtained by adding a number of corrections to the experimental temperature rise as

$$\Delta T_{\text{ideal}}=\Delta T_{\text{experimental}}+\sum_{i}\delta T_{i}.$$ (4)

These temperature rise corrections are described in references [2,4]. Our implementation of the corrections follows these two references with the exception of the thermal radiation correction. This correction is dependent on the optical properties of the fluid and the cell, and is discussed in more detail below.

### 2.1 The Radiation Correction

If the fluid is transparent to infrared radiation, then this correction is only a function of the cell geometry and the optical properties of the materials used in its construction. The radiation correction described in references [2,4] assumes that all of the surfaces in the cell are blackbodies. The blackbody radiation correction is given by

$$\delta T_{5T}=\frac{8\pi r_{0}\sigma T_{0}^{3}\Delta T^{2}}{q}$$ (5)

where σ is the Stefan-Boltzmann constant. In practice, many experimenters assume that this correction is negligible and neglect the correction. We have found that this correction changes the reported thermal conductivity of argon at 300 K by about 1% for our geometry, so it is not appropriate to ignore it. A more accurate correction can be obtained by considering the optical properties of the surfaces in the hot-wire cell.

For this analysis we consider the cell surfaces to be diffuse gray surfaces and follow the analysis presented in reference [5]. We consider the cell to be an infinitely long hot wire in a concentric cylindrical cavity. Thus, two surfaces are involved in the heat transfer. Surface 1 is the hot wire whose temperature is a function of time, and surface 2 is the cylindrical cavity surrounding the hot wire which remains at the initial equilibrium temperature. The net radiative heat flux for the hot wire, using the tabulated view factors in reference [5], is

$$Q_{1}=\frac{A_{1}\sigma \left(T_{1}^{4}-T_{2}^{4}\right)}{\frac{1}{\epsilon_{1}}+\frac{A_{1}}{A_{2}}\left(\frac{1}{\epsilon_{2}}-1\right)},$$ (6)

where *A_i_* is the area, *T_i_* is the temperature, and ϵ*_i_* is the emissivity of surface *i*. The ratio of the surface areas *A*_1_/*A*_2_ which is present in the denominator of eq (6) is quite small since very thin hot wires are used. In our cell this surface area ratio is A_1_/*A*_2_=0.001. The inverse emissivity of the hot wire 1/ϵ_1_ varies from 10 to 25 for platinum and l/ϵ_2_ is approximately 2. Therefore, the second term in the denominator of eq (5) is negligible to within 0.1% in *Q*_1_, and we are left with

$$Q_{1}=A_{1}\epsilon_{1}\sigma \left(T_{1}^{4}-T_{2}^{4}\right).$$ (7)

Because the surface area of the cavity surrounding the hot wire is so much larger than the surface area of the hot wire, to a first approximation the heat transfer is not a function of the emissivity of the cavity.^2^ The cavity appears to be a blackbody, and the heat transfer is only a function of the emissivity of the platinum hot wire. Following the analysis of reference [4], the resulting correction to the experimental temperature rise in a transparent fluid is

$$\delta T_{5T}=\frac{8\pi r_{0}\epsilon_{\text{platinum}}\sigma T_{0}^{3}\Delta T^{2}}{q}.$$ (8)

The emissivity of platinum, ϵ_platinum_, is a function of temperature and is tabulated in reference [6]. At 300 K the emissivity of platinum is 0.0455 relative to an emissivity of 1 for a blackbody. The black-body radiation correction of eq (5) is roughly 20 times larger than the real case, eq (8), when platinum hot wires are used.

For fluids which absorb infrared radiation, the technique described in reference [7] works well. The technique is based on the numerical simulations of transient conduction and radiative heat transfer from a hot wire in an absorbing medium. Since the emissivity of the platinum hot wire is so small, the radiative heat flux from the wire is negligible in the simulations. The primary mechanism for radiative losses is from emission from the fluid at the boundary of the expanding conduction front. This analysis [7] yields a radiation correction for absorbing media which is given by

$$\delta T_{5A}=-\frac{qB}{4\pi \lambda }\left[\frac{r_{0}^{2}}{4a}\ln\left(\frac{4at}{r_{0}^{2}C}\right)+\frac{r_{0}^{2}}{4a}-t\right].$$ (9)

The radiation parameter *B* is related to the fluid properties by

$$B=\frac{16Kn{\;}^{2}\sigma T_{0}^{3}}{{\rho C}_{p}},$$ (10)

where *K* is the mean extinction coefficient of the fluid and *n* is its refractive index. These fluid properties are a function of the fluid density and temperature and are not generally available. The procedure described in reference [7] allows *B* to be estimated from the experimental temperature rise data. Equation (9) indicates that the radiation correction introduces a term which is a direct function of time into the temperature rise equation. When the radiation correction is added to the ideal temperature rise, we obtain

$$\Delta T=\frac{q}{4\pi \lambda }\left[1+\frac{Br_{0}^{2}}{4a}\right]\ln\left(\frac{4at}{r_{0}^{2}C}\right)-\frac{Bqt}{4\pi \lambda }+\frac{Bqr_{0}^{2}}{16\pi a\lambda }+\ldots .$$ (11)

Thus, we correct the experimental data with all the other corrections and fit the resulting temperature rise to a function of the form

$$\Delta T=C_{1}\ln\left(t\right)+C_{2}t+C_{3}.$$ (12)

The experimental radiation parameter *B* is determined from coefficient *C*_2_ using

$$B=C_{2}\left(\frac{-4\pi \lambda }{q}\right).$$ (13)

Once *B* is determined, we use eq (9) to correct for radiation in the absorbing fluid. This technique allows us, as shown later, to use our experimental data to determine whether there is a significant thermal radiation correction in an absorbing fluid and to correct for the radiation. No prior knowledge of the optical properties of the fluid is required.

## 3. Apparatus

The apparatus is quite similar to a previously described low temperature system [8] which is used from 80 to 320 K. The new apparatus is designed to operate from 220 to 750 K at pressures to 70 MPa. A preliminary version of the new instrument has been described elsewhere [9]. Improvements have been incorporated into the new system to improve the precision and accuracy of the thermal conductivity measurement and to enable measurement of the thermal diffusivity. They were based on modifications introduced in the low temperature system which are fully described in references [10] and [11].

### 3.1 Hot Wires

**Figure 1:**
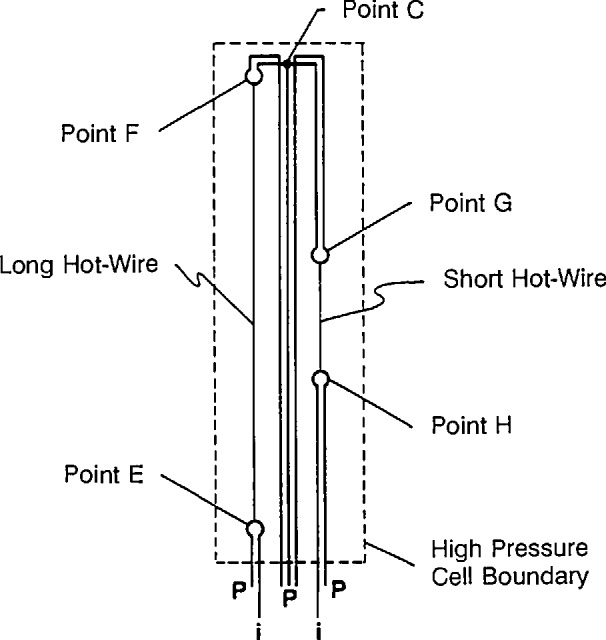
Arrangement of current leads (i) and potential taps (P) within the pressure cell. Bridge points correspond to those in figure 3.

The hot wires are selected to conform to the ideal line-source model as closely as possible. The line-source model assumes that the wire has no heat capacity and that it is infinitely long, so there is no axial heat conduction. The wire diameter is 12.7 µm in this instrument to minimize effects due to its finite heat capacity while retaining good tensile strength and uniformity. A two-wire compensating system is used in order to eliminate effects due to axial heat conduction. The arrangement of the two wires is shown in figure 1. The two wires have different lengths and are arranged in a modified Wheatstone bridge where the thermal response of the short wire is subtracted from the response of the long wire. The resulting response from a finite length of wire approximates that of an infinitely long hot wire. The length of the equivalent wire is the difference in the lengths of the long and short hot wires.

The hot wires are used simultaneously as electrical heat sources and as resistance thermometers. Platinum wire is used in this instrument because its mechanical and electrical properties are well known over a wide temperature range, and it is resistant to corrosion up to 750 K. As shown above, platinum has the added advantage of low emissivity. The length of the long hot wire is about 19 cm. The length of the short hot wire is about 5 cm. The platinum hot wires are annealed after they are installed, so that their resistance will be stable during high temperature operation. The resistance of the annealed hot wires is about 20% less than the hard-drawn platinum wire. The resistance of the hot wires is calibrated *in situ* as a function of temperature and pressure [12].

The wires are welded to rigid upper suspension stirrups and weighted lower suspension stirrups. The floating lower weights are used to tension the wires and to allow for thermal expansion. There are fine copper wires welded between the bottom weights and the massive bottom leads. These fine wire leads are flexible so that they do not introduce significant stress on the platinum hot wires. This arrangement provides both current and potential leads to both ends of each hot wire. Thus, four-terminal resistance measurements can be made on both the long and short hot wires, eliminating uncertainty due to lead resistances.

### 3.2 Hot-Wire Cell

The two platinum hot wires are contained in a pressure vessel which is designed for 70 MPa at 750 K. The cell is connected with a capillary tube to a sample-handling manifold. This sample-handling manifold allows evacuation of the cell, charging and pressurization of liquids with a screw pump, and pressurization of gases with a diaphragm compressor. There are seven electrical leads into the pressure vessel to enable four-terminal resistance measurements of both hot wires. The electrical leads pass through a 6.25 mm O.D. pressure tube which connects the bottom of the pressure vessel to the lead pressure seal. The pressure seal for the electrical leads is made at ambient temperature for improved reliability. The vessel access tube is located on the bottom of the vessel so that there is always a positive temperature gradient with respect to height to eliminate free convective driving forces. The entire pressure system is constructed of 316 stainless steel for corrosion resistance.

**Figure 2:**
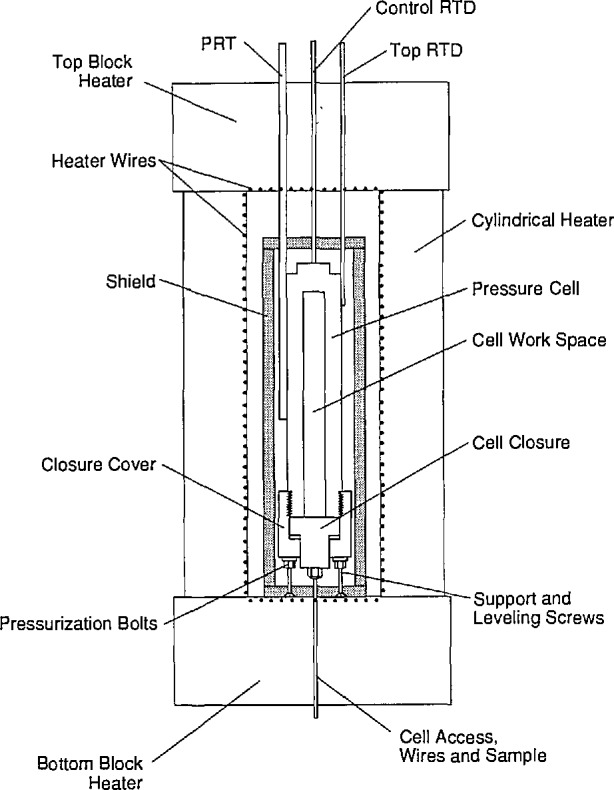
High-pressure cell, shield, and furnace.

The thermal conductivity cell is shown in its temperature control environment in figure 2. The cell pressure vessel is surrounded by a 12 mm thick cylindrical aluminum heat shield. The aluminum has a high thermal conductivity and provides a nearly isothermal environment for the pressure vessel. There is an air gap between the vessel and the heat shield. This air gap isolates the pressure vessel from temperature fluctuations in the heat shield. Tubes are silver-soldered to the outside of the pressure vessel which enclose the reference standard platinum resistance thermometer (PRT) and two smaller platinum resistance probes (RTDs). The two RTDs can be moved axially along the vessel to detect temperature gradients. Normally, one RTD is located near the top of the vessel, and the other RTD is located near the bottom of the vessel. This configuration allows us to measure the cell temperature at the center of the vessel with the reference standard PRT and temperature gradient in the cell with the two RTDs for each thermal conductivity measurement.

For experiments from ambient temperature to 750 K, the vessel and heat shield are placed in a cylindrical furnace constructed of heating elements cast in fibrous ceramic insulation. These heating elements are shown in figure 2 and are separated from the aluminum heat shield by a second air gap. An additional platinum RTD is located on the top of the aluminum heat shield. This probe provides the feedback signal for the furnace temperature control system. The main power supply is under computer control and is connected to the bottom end heating element and the tubular heating elements. The second trim power supply is manually controlled to eliminate axial gradients in the thermal conductivity cell. The heating elements are driven with dc power supplies to minimize electromagnetic noise in the thermal conductivity instrument. Temperature fluctuations in the cell are normally less than 0.01 K.

For experiments between 220 and 300 K, the electrical heaters are replaced by a copper cooling coil enclosed in polystyrene insulation. A refrigerant with a low freezing point is pumped through the cooling coil by a recirculating temperature control bath. This recirculating bath controls the fluid temperature to within 0.01 K. The aluminum heat shield and air gap further reduce the temperature fluctuations in the cell to less that 0.01 K.

### 3.3 Wheatstone Bridge Circuit

**Figure 3:**
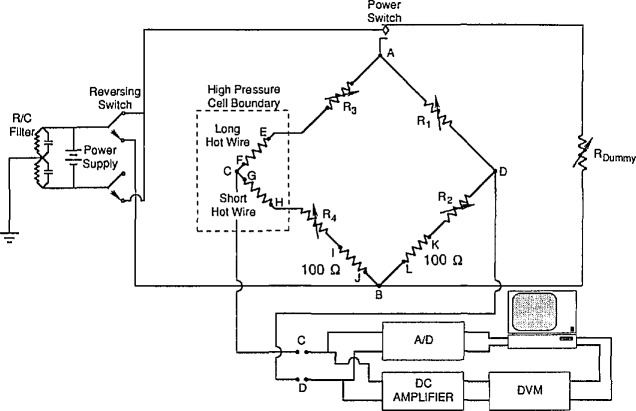
The Wheatstone bridge schematic for the transient hot-wire apparatus. Potential taps are indicated by points A–L.

This instrument uses a Wheatstone bridge circuit to monitor the resistance changes of the hot wires during the step-power pulse. The two hot wires are set up in opposing legs of the Wheatstone bridge as shown in figure 3. The drive voltage is applied across points A and B. The bridge response is monitored by a high speed digital multimeter across points C and D. The bridge is initially balanced with a 100 mV drive voltage. There is negligible heating of the hot wires with this small balance voltage. The four legs of the Wheatstone bridge are designated Rl, R2, R3, and R4. Each of the four legs contains a variable decade resistor. The smallest step on these decade resistors is 0.01 Ω. These four decade resistors are adjusted so that the bridge imbalance signal is 0 and the total resistance of each leg is the same.

There are two current paths between points A and B. Each current path contains a calibrated 100 Ω standard resistor in order to determine the current flowing through that path during the balancing procedure. Figure 3 shows a number of voltage taps on the Wheatstone bridge which allow the multiplexed digital multimeter to measure the voltage drops across all of the resistances in the bridge. Using the current, provided by the voltage drop across the standard resistors, we can obtain the resistance of all of the components of the bridge.

These resistances must be known very precisely, and the bridge must be balanced very closely, in order to obtain accurate thermal diffusivities from the experiment. Thermal voltages from the components of the bridge have a significant impact on the balancing of the bridge. In order to eliminate errors from thermal voltages, the bridge is alternately measured with a positive and negative drive voltage with a reversing relay. During the balancing procedure, 10 alternating drive voltage cycles are measured. During each cycle the digital multimeter monitors the voltage across all of the voltage taps. These values are subsequently averaged and displayed by the system computer.

When a satisfactory bridge balance is obtained, we are ready to begin the transient hot-wire experiment. The power supply is switched to a dummy resistor and the drive voltage is set to a level which will produce the desired heating of the hot wires. The experiment begins when the power supply is switched from the dummy resistor to the Wheat-stone bridge. During the experiment the multimeter records the bridge voltage as a function of time across points C and D. This signal is proportional to the differential resistance change of the two hot wires. This differential resistance change of the two wires is related to the temperature changes of the two hot wires by the wire calibration which is described below. The experiment normally lasts 1 s with a bridge response voltage recorded every 4 ms.

### 3.4 Data Acquisition and Control

Data acquisition and control are coordinated by a personal computer. The computer controls the cell temperature, synchronizes the experimental timing, records the data, and provides a graphical display of the data. The computer has an analog-to-digital interface board which generates the timing signals based on the computer’s internal quartz crystal oscillator and controls the system voltage multiplexers. The computer is also equipped with an IEEE-488 interface which allows communication with a dedicated digital temperature controller, a digital nanovoltmeter, and the high speed digital multimeter.

The cell PRT and the two gradient RTDs are connected in series with a standard resistor and a precision 1 mA current source. The computer controls a multiplexer which allows the nanovoltmeter to measure the voltage drops across the three resistance thermometers and the calibrated standard resistor. Using the current which is determined by the voltage drop across the standard resistor, we can obtain the resistances of the three thermometers.

A second multiplexer is connected to the input of the high speed digital multimeter. This multiplexer allows sampling of all the voltage taps on the Wheatstone bridge during bridge balancing. Since standard resistors are included in both current paths of the bridge, we can obtain accurate measurements of all the resistances in the bridge. The resistance of the two hot wires is used in conjunction with the PRT temperature to obtain the calibrations for the hot wires. In addition, the multiplexer allows us to measure the drive voltage and the resistance of the power switching relay for an accurate determination of the power applied to the hot wires.

During the experiment, there are two parallel systems measuring the bridge response. A 16 bit analog-to-digital converter directly monitors the bridge response, while the high speed digital multimeter monitors the response of an instrumentation amplifier which is also connected across points C and D. The instrumentation amplifier has a fixed gain of 100 and also has an analog filter built in. This filter significantly reduces the noise of the bridge response but introduces a time lag which we must account for. The noise of the raw signal is 25 μV but is reduced to 3 μV by the filter. The experimental timing is fixed by the raw signal which is monitored by the analog-to-digital converter. The relatively noisy raw signal is used to adjust the timing of the filtered bridge response which is recorded by the high speed digital multimeter.

## 4. Hot-Wire Calibration

The electrical resistance of pure platinum as a function of temperature is very well characterized because of its widespread use in thermometry. In most thermometry applications the platinum is maintained at ambient pressure. In transient hotwire instruments, however, the platinum is immersed directly in the fluid of interest. Roder et al. [12] showed that the effect of pressure on the resistance of the platinum hot wires must be accounted for. The functional form of our calibration is given by

$$R\left(T,P\right)=A+BT+CT^{2}+\left(D+ET\right)P,$$ (14)

where *R* is the wire resistance, *T* is the temperature, and *P* is the applied pressure.

We have found that an *in situ* calibration provides the most reliable measurements possible. In practice, we obtain the resistance of both hot wires at the cell temperature and pressure for every experiment. The calibration process is an integral part of balancing the bridge. As described above, we have the capability to make a four-terminal resistance measurement of each hot wire without errors from the temperature-dependent lead resistance. When we have completed all measurements on a given fluid, we do a surface fit of the resistance of each wire using the functional form above. Examining trends in deviations from this surface fit helps us to detect inconsistent data. Slow changes in the calibration usually indicate changes in the physical condition of the hot wires, such as continued annealing of the platinum at high temperatures. Sudden changes in the wire calibration provide an indication of mechanical damage to the wires. In addition, the capability to generate an *in situ* calibration provides freedom to use materials other than platinum for the hot wires.

## 5. Performance Verification

Toluene was selected to verify the instrument performance in the liquid phase since it has been recently recommended as a thermal conductivity reference standard [13]. Argon and nitrogen were selected to verify performance of the apparatus in the gas phase since they have been widely studied with both steady-state techniques and transient hot-wire instruments. In addition, they have been studied with our low temperature instrument so that discrepancies between the two instruments can be detected and resolved.

### 5.1 Toluene

The thermal conductivity of liquid toluene has been widely studied with both steady-state and transient hot-wire instruments for a number of years. Early steady-state experiments on toluene were often plagued by free convection. Free convection is easily avoided in a transient hot-wire instrument, but, if present, is easily detected due to deviations from the ideal line-source model. The contribution of thermal radiation to the apparent thermal conductivity of toluene has also been of much concern since toluene is not transparent in the infrared. Nieto de Castro et al. [7] have made an extensive study of thermal radiation and concluded that the radiative contribution to heat transfer is very small for toluene at temperatures up to 370 K. Above 370 K, it was estimated that the contribution of heat transport by radiation to the measured value of thermal conductivity would increase with temperature resulting in nonzero values of the quantity *B* in eq (13). Toluene was selected to verify both the performance of the new instrument in the liquid phase and the size and effect of the radiative contribution at the higher temperatures.

The spectroscopic grade toluene used in our verification measurements was dried over calcium hydride and distilled to remove a trace of benzene impurity. The purified toluene was analyzed by gas chromatography and found to have less than 50 parts per billion (ppb) benzene and less than 100 ppb water. The results of the saturated liquid toluene tests are provided in table 1. In order to obtain the isobaric heat capacity from the measured thermal diffusivity, we have calculated the density with the equation of state of Goodwin [14].

**Figure 4:**
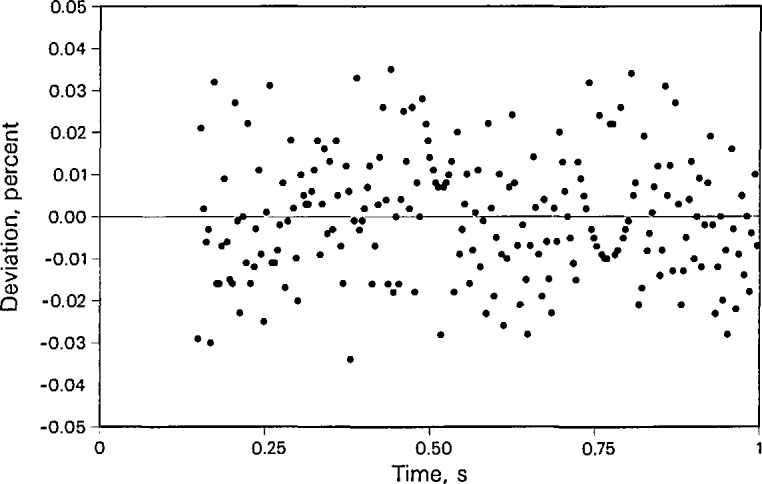
Typical deviations of experimental temperature rises from the calculated straight line versus the log of time for liquid toluene data point 1202 at *T*_0_ = 324.039 K and *P* = 0.088 MPa.

Figure 4 shows a typical deviation plot of the experimental temperature rises from the full heat transfer model for a liquid phase toluene point (number 1202) at a temperature of 324 K. The deviations from linearity are less than 0.04%. The deviations show that much of the noise is due to 60 Hz electromagnetic interference, but the noise is acceptably small. Table 1 shows two additional statistics which reflect nonlinearity of each data set relative to the ideal line source model, eq (1), after correcting according to eq (4). The first term is “STAT” which reflects the uncertainty in the slope of the regression line at a confidence level of 2 times the standard deviation (2σ). The term “DSTAT” reflects the uncertainty in the intercept of the regression line at a 2σ confidence level. For instance, a value of “STAT” or “DSTAT” of 0.001 indicates the 2σ uncertainty is 0.1%. As discussed earlier, we expect the thermal radiation correction to affect the measured thermal conductivity of toluene more and more as the temperature is increased above 370 K. The effect can be seen in the statistic “STAT” which is a numerical description of a deviation plot such as figure 4. Graphically, the deviation plots are no longer random but become systematically curved, as predicted by eq (11). Consequently, the thermal conductivities obtained from the usual linear fit are larger than they should be. To obtain correct results, we apply eq (12) to the experimentally measured temperature rises and evaluate *B* for every individual point. Next, the experimentally determined values for *B* are fit to a linear function in temperature. The resulting expression is

$$B=-0.0685+2.310\times 10^{-4}T_{0}$$ (15)

where *B* is in s^−1^ and *T*_0_ is in K. The values given by eq (15) are used to re-evaluate the radiation correction, δ*T*_5_, for each data point. The results corrected in this fashion are given in table 1.

**Figure 5:**
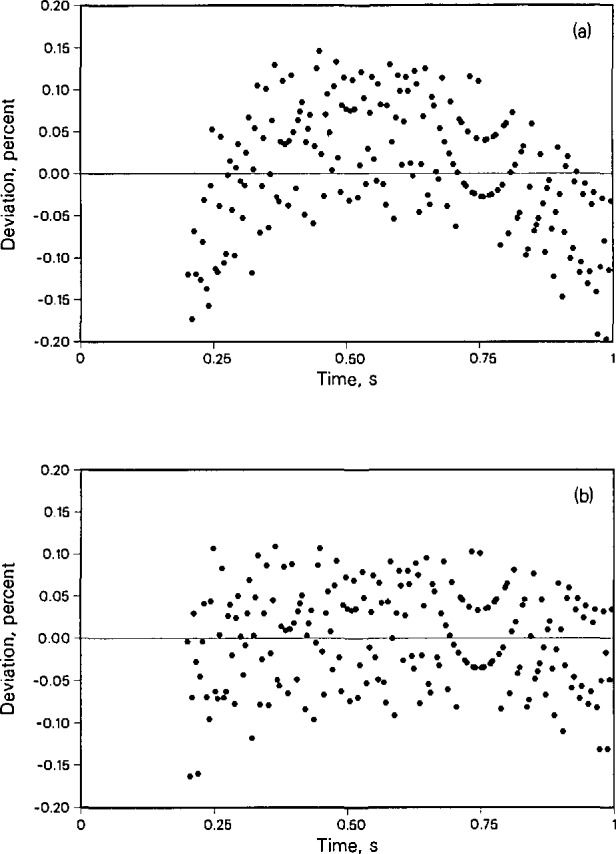
Liquid toluene data point 2105 at *T*_0_ = 548.140 K and *P* = 2.686 MPa. a) before application of the radiation correction, eq (9), “STAT” is 0.002. b) after application of the radiation correction, eq (9), “STAT” is 0.001.

Figure 5 shows the deviation plot for the temperature rises for a toluene data point (2105) at *T*_0_ = 548.140 K and *P* = 2.686 MPa, before and after the radiation correction δ*T*_5_ has been applied. The deviation “STAT” has decreased from 0.002 to 0.001 and the curvature has been eliminated. These results support the model developed by Nieto de Castro et al. [7] to account for the effect of radiation in absorbing media, and suggest that the instrument with a revised δ*T*_5_ is operating in accordance with its mathematical model.

**Figure 6:**
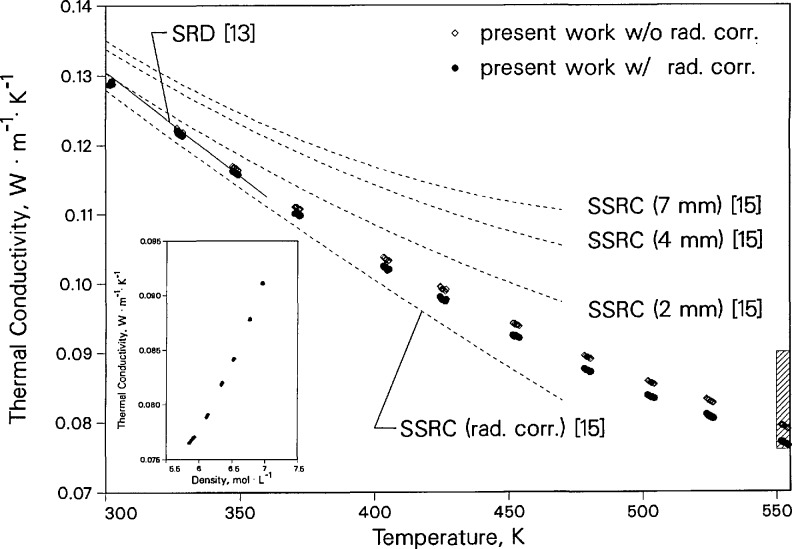
The thermal conductivity of liquid toluene near the saturation line. Dashed lines show steady-state rotating-cylinder (SSRC) results at various spaeings along with the extrapolated radiation-corrected results. Solid line is the SRD correlation. The inset represents the thermal conductivity of toluene as a function of density near 550 K. The region of the inset is shaded on the main figure.

Figure 6 shows both the uncorrected and the radiation corrected thermal conductivity values of toluene near the saturation line as a function of temperature. The standard reference data correlation of Nieto de Castro et al. [13], which is valid to 360 K, is a line shown in figure 8. The measurements of Fischer and Obermeier [15] are also displayed. These were obtained with a rotating concentric-cylinder apparatus, operating in steady-state mode, for different gaps between the cylinders. We have included their extrapolation to zero gap, which is considered to be their radiation-corrected thermal conductivity. Figure 6 shows that our transient hot-wire instrument has a smaller radiation contribution than the steady-state measurements. However, the transient hot-wire radiation contribution becomes significant at elevated temperatures, 3.1% at 550 K. The larger radiation contribution in steady-state methods produces much larger uncertainty in the extrapolated radiation-corrected thermal conductivity data obtained with steady-state instruments. The temperature dependence along the saturation boundary, shown in figure 6, is similar to the trend reported in reference [13] with respect to the thermal conductivity data of Nieto de Castro et al. [7]. The data above 370 K show the presence of radiative effects. Also shown in figure 6, as an insert, are the compressed-liquid data at 550 K, which correspond to the shaded area of the diagram.

**Figure 7:**
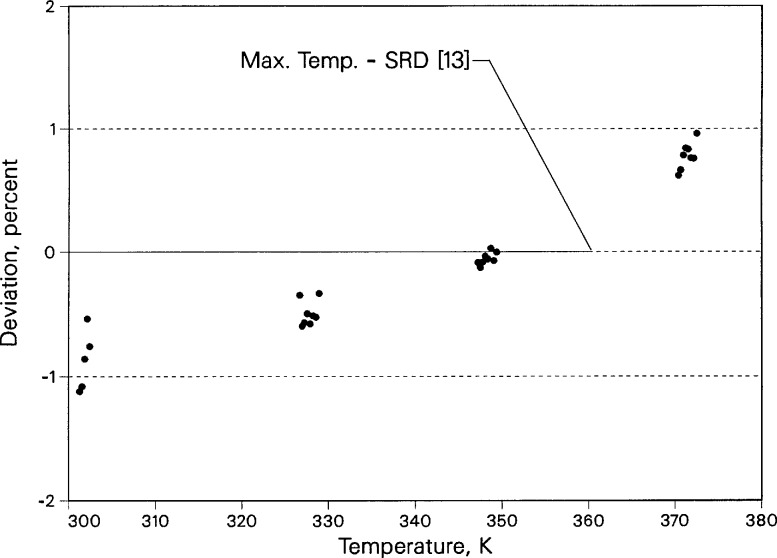
Deviation of liquid toluene thermal conductivity near saturation pressure relative to the correlation of Nieto de Castro et al. [13].

Deviations between the toluene thermal conductivity data and the correlation by Nieto de Castro et al. [13] are shown in figure 7 for temperatures up to 380 K. All of the data are within 1% of the correlation from 300 to 372 K; however, the deviations are systematic. We suggest that a higher-order temperature-dependent term might be added to the correlation in order to extend its temperature range.

**Figure 8:**
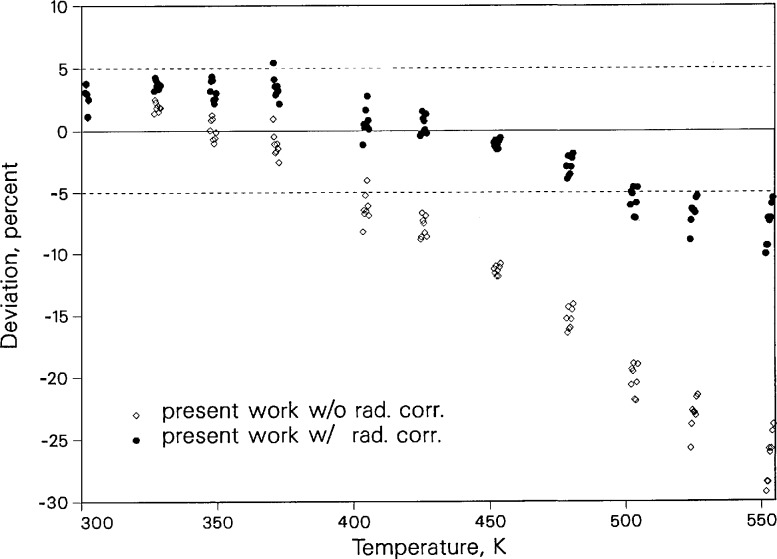
Deviation of liquid toluene isobaric heat capacity near saturation pressure relative to the *C_p_* calculated using the equation of state of Goodwin [14].

Figure 8 displays the deviations between the heat capacity of toluene obtained from the measured thermal diffusivity and thermal conductivity using the density from the equation of state of Goodwin [14], versus the *C_p_* value calculated by this equation of state. The data, uncorrected for radiation, show systematic departures from the equation-of-state prediction above 370 K, with deviations of 30% at 550 K. After the adjusted radiation correction δ*T*_5_ is applied, the deviations decrease to less than 10% at the highest temperature, falling in a band of ±5% up to 500 K. The larger deviations above 500 K are still within the combined uncertainties of our diffusivity measurements and the equation of state of Goodwin [14].

Figures 6 and 8 demonstrate the performance of the instrument for the measurement of both thermal conductivity and thermal diffusivity at high temperatures in infrared absorbing fluids when the radiation correction, given by eqs (9) to (13) and (15), is applied.

### 5.2 Argon

We have previously reported two sets of transient hot-wire measurements of argon’s thermal conductivity near 300 K [16,17]. Both of these data sets were made with the low temperature instrument described by Roder [8]. Thermal conductivity measurements on argon have also been reported by a number of other researchers [18–22]. Table 2 provides the results for the present measurements near 300 K. Younglove’s equation of state [23] is used to obtain the densities reported in the table 2. The purity of the argon used in these measurements is better than 99.999%. Argon is transparent to thermal radiation, and the radiation correction at 300 K is negligible.

**Figure 9:**
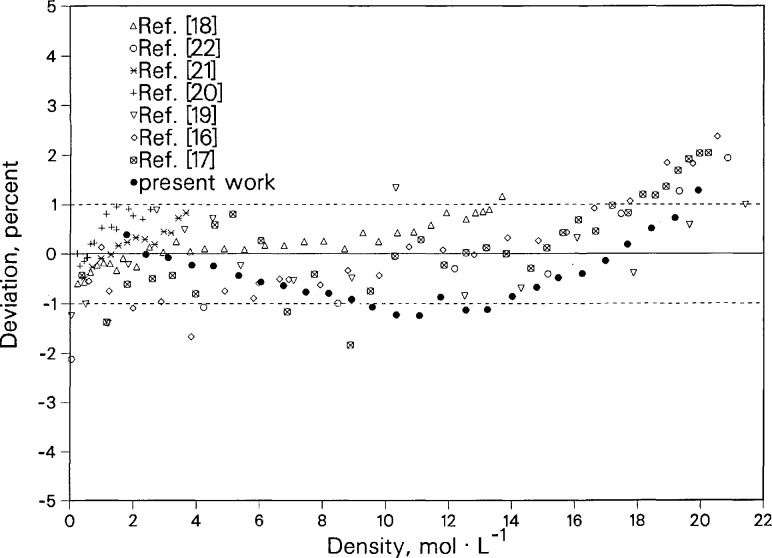
Deviations of argon thermal conductivity data near 300 K relative to the correlation of Perkins et al. [24].

Deviations between the present thermal conductivity data and the new surface fit of Perkins et al. [24] as a function of density are shown in figure 9. The maximum deviation between our present measurements and the correlation is 1.2% at the highest densities. The present data were not, however, used in the development of the thermal conductivity surface [24]. The same trend of deviations relative to the correlation is exhibited by the other available data. Our thermal conductivity data agree with the results of the other data within ± 1%. All of the other data were made with transient hotwire instruments, with the exception of data from Michels et al. [19], which was obtained with a steady-state parallel-plate instrument.

### 5.3 Nitrogen

For the present instrument, table 3 provides the results on nitrogen for temperatures near 425 K. Younglove’s equation of state [23] is used to obtain the densities reported in the table 3. The purity of the nitrogen used in these measurements is better than 99.999%. Nitrogen is transparent to thermal radiation, and the radiation correction at 425 K is negligible.

**Figure 10:**
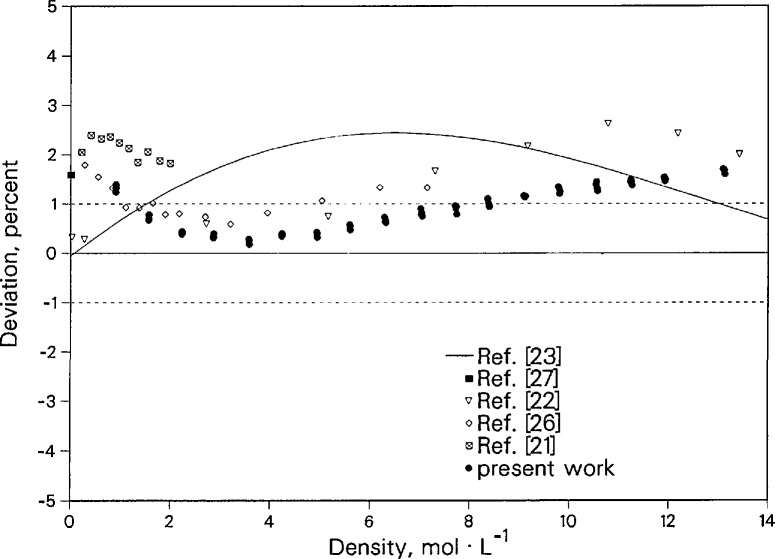
Deviations of nitrogen thermal conductivity near 428 K relative to the correlation of Stephan et al. [25].

Deviations between our thermal conductivity data and the correlation of Stephan et al. [25] as a function of density are shown in figure 10. The maximum deviation between our measurements and the correlation is 2%. Nitrogen thermal conductivity measurements have also been reported by several other researchers [21,22,26]. The same trend of deviations relative to the correlation is exhibited by the other available data. Our thermal conductivity data agree with those results to 1%, except for values from reference [22] for densities above 9 mol·L^−1^. All of the other data were obtained with transient hot-wire instruments, with the exception of data from le Neindre [22], which were obtained with a steady-state concentric-cylinder instrument. The dilute gas value of Millat and Wake-ham [27] is also plotted in this figure and agrees with the extrapolation of the present data within 0.5%. There is both theoretical [27] and experimental [28] evidence that the low density values of the Stephan et al. correlation [25] need to be revised. The correlation given by Younglove [23] has a completely different curvature as already shown in reference [28].

**Figure 11:**
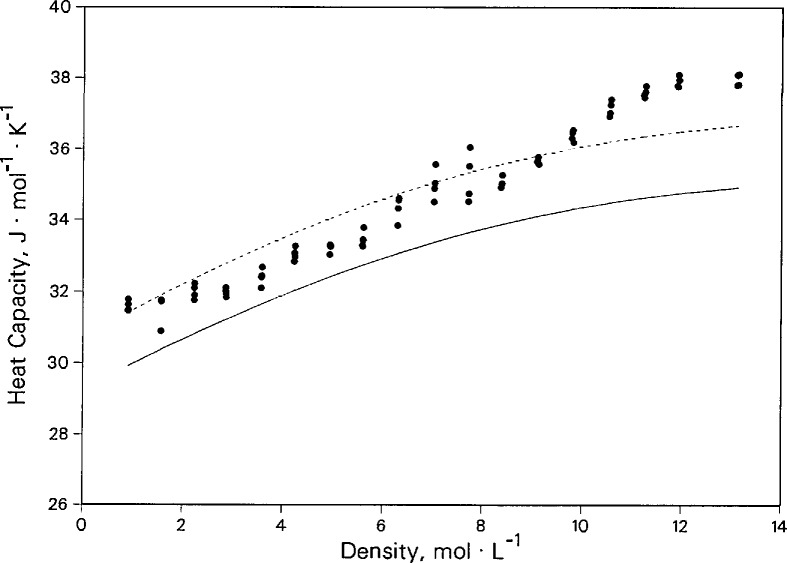
Nitrogen isobaric heat capacity relative to values calculated (solid line) from the equation of state of Younglove [23]. Dashed line is a 5% offset from [23].

Figure 11 shows heat capacities of nitrogen given in table 3 for the isotherm at 425 K. The values are derived from the measured values of thermal conductivity and thermal diffusivity taking the densities from the equation of state [23]. They are compared to values calculated from the equation of state, and they are systematically higher than the equation-of-state predictions by about 5% except for the highest densities. We assign an estimated error of ±5% to our measured heat capacities; the error estimated for the specific heats from the equation of state is also 5%. Thus, the agreement between the two sources is within their mutual uncertainties even at the higher densities.

### 5.4 Repeatability Tests

**Figure 12:**
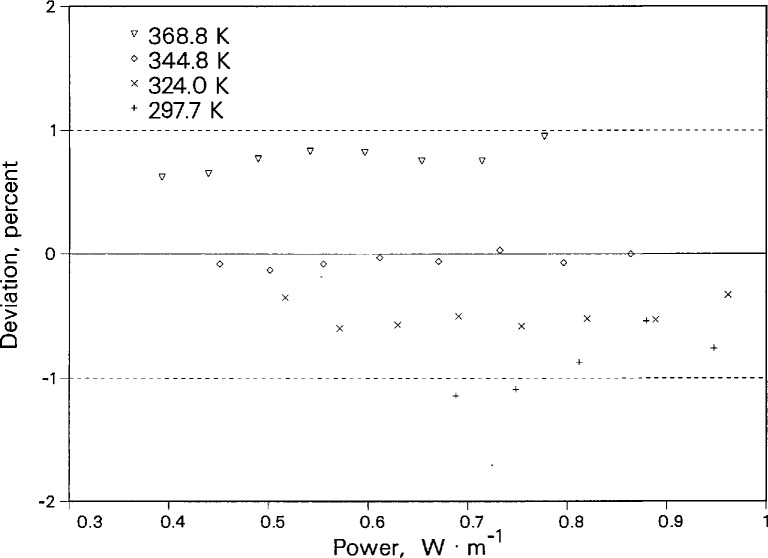
Deviations in the thermal conductivity of liquid toluene as a function of applied power. Baseline is the correlation of Nieto de Castro et al. [13]. Dashed lines show 95% uncertainty band.

In addition to comparisons of our thermal conductivity data with the data and correlations of other researchers, we have made many measurements to assess the repeatability of the instrument. The temperature assigned to a given thermal conductivity measurement is a function of the fluid temperature rise during the experiment. As a result, each power represents a different and independent temperature rise and experimental temperature. For a given cell temperature, we routinely make measurements at many powers not only to verify the instrument performance but also to check on the presence of convection. To check repeatability, results at different powers are compared in terms of deviations from a correlation of the thermal conductivity surface. Figure 12 shows deviations of the liquid toluene thermal conductivity data for four cell temperatures as a function of the applied power. There are from five to eight different powers for each cell temperature. The maximum difference between the deviations for each cell temperature is about 0.3%, which is equivalent to the experimental precision in λ. The deviations do not appear to have any power dependence.

**Figure 13:**
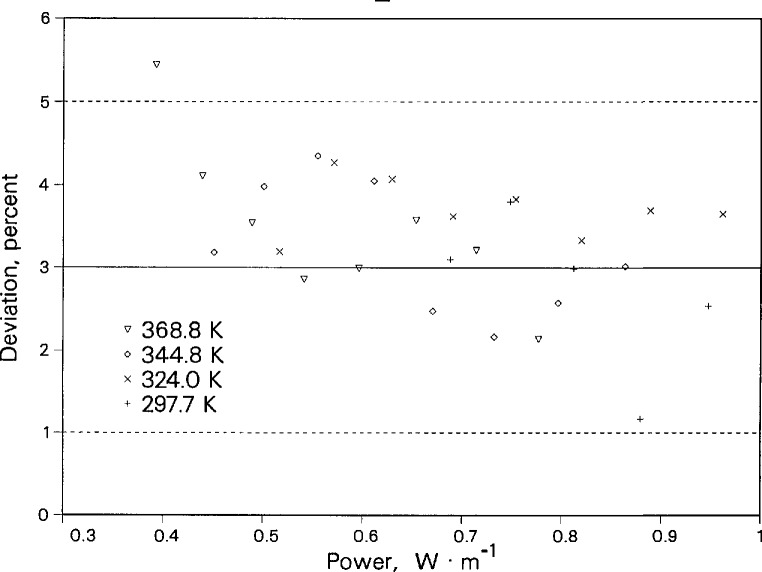
Deviations in the isobaric heat capacity of liquid toluene as a function of applied power. Baseline is the equation of state of Goodwin [14]. Dashed lines show 95% uncertainty band.

The power dependence of the isobaric heat capacity of liquid toluene is shown in figure 13. The maximum difference between the deviations for each temperature is 2.6%. Again there is no discernable trend in the deviations of the heat capacity with respect to the applied power.

**Figure 14:**
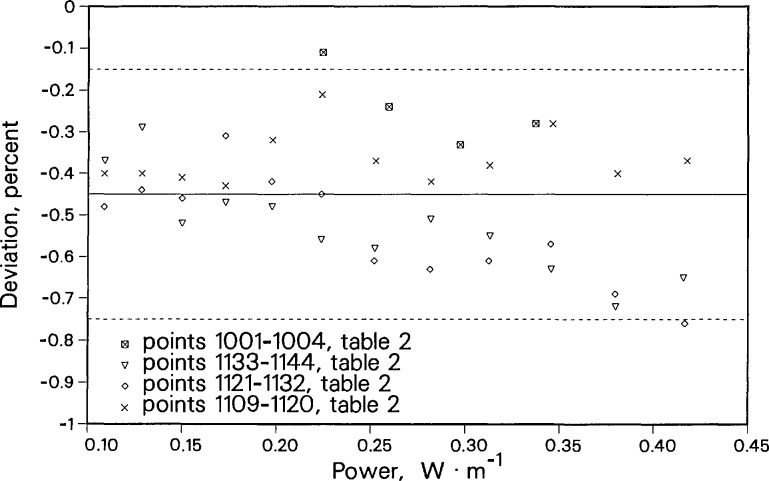
Deviations in the thermal conductivity of argon gas as a function of applied power. Baseline is the correlation of Younglove et al. [29]. Dashed lines show 95% uncertainty band.

Figure 14 shows a deviation plot of 40 argon thermal conductivity data points relative to the correlation of Younglove et al. [29]. The applied power ranges from 0.11 to 0.42 W/m for a range of final temperature rises from 0.8 to 5 K. The data were obtained in four different sequences over 2 days. The four measurement sequences are shown with different plot symbols. The deviations from the correlation range from about −0.1% to −0.7%. Thus, the set of 40 measurements are consistent with each other and fall within a band of ±0.3%. The instrument’s response is shown to be independent of applied power over a very wide range of temperature rises. The instrument’s performance is also very repeatable over an extended period.

## 6. Summary

A new transient hot-wire thermal conductivity instrument for use at high temperatures is described. This instrument has an operating range from 220 to 750 K at pressures to 70 MPa. Thermal conductivity can be measured over a wide range of fluid density, from the dilute gas to the compressed liquid. The thermal conductivity data have a precision of ±0.3% and an accuracy of ±1%. The instrument is also capable of measuring the thermal diffusivity with a precision of ± 3% and an accuracy of ±5%. Given accurate fluid densities, we can obtain isobaric heat capacities from the data. This instrument complements our low temperature instrument [8] which has a temperature range from 80 to 325 K at pressures to 70 MPa. A detailed analysis of the influence of radiative heat transfer in the transient hot-wire experiment has been performed, and radiation-corrected thermal conductivities are reported for liquid toluene near saturation at temperatures between 300 and 550 K. In addition, new measurements of the thermal conductivity and thermal diffusivity of argon and nitrogen verify the performance of the apparatus.

## Figures and Tables

**Table 1:** Thermal conductivity, thermal diffusivity, and heat capacity of liquid toluene from 300 to 550 K

Run Pt.	Pressure MPa	Temperature K	Density mol/L	Power W/m	Thermal conductivity W/(m·K) STAT	Cell temperature K	Thermal diffusivity m^2^/s DSTAT	Specific heat J/(mol·K)
1101	0.090	302.441	9.3100	0.94731	0.12880 0.000	297.811	0.866×10^−7^ 0.003	160.6
1102	0.090	302.128	9.3131	0.87943	0.12918 0.000	297.840	0.880×10^−7^ 0.003	158.4
1103	0.090	301.835	9.3160	0.81210	0.12885 0.000	297.872	0.861×10^−7^ 0.004	161.3
1104	0.090	301.549	9.3188	0.74835	0.12865 0.000	297.869	0.852×10^−7^ 0.004	162.7
1105	0.090	301.265	9.3215	0.68819	0.12868 0.001	297.891	0.858×10^−7^ 0.005	161.5
1201	0.088	328.877	9.0466	0.96177	0.12145 0.000	323.969	0.791×10^−7^ 0.003	170.8
1202	0.088	328.528	9.0501	0.88883	0.12132 0.000	324.039	0.789×10^−7^ 0.003	170.9
1203	0.088	328.174	9.0537	0.81994	0.12144 0.000	324.005	0.792×10^−7^ 0.004	170.2
1204	0.088	327.841	9.0571	0.75378	0.12146 0.000	323.979	0.788×10^−7^ 0.004	171.1
1205	0.088	327.506	9.0605	0.69063	0.12166 0.001	323.937	0.790×10^−7^ 0.005	170.7
1206	0.089	327.180	9.0638	0.62968	0.12167 0.001	323.951	0.786×10^−7^ 0.006	171.5
1207	0.088	326.927	9.0664	0.57154	0.12171 0.001	323.951	0.784×10^−7^ 0.007	1715
1208	0.088	326.638	9.0693	0.51693	0.12210 0.001	323.927	0.795×10^−7^ 0.007	170.0
1301	0.086	347.212	8.8572	0.45110	0.11626 0.001	344.798	0.744×10^−7^ 0.008	177.1
1302	0.086	349.371	8.8346	0.86369	0.11571 0.000	344.881	0.745×10^−7^ 0.003	176.8
1303	0.086	349.037	8.8381	0.79636	0.11573 0.000	344.778	0.748×10^−7^ 0.003	176.0
1304	0.086	348.691	8.8417	0.73227	0.11595 0.000	344.785	0.752×10^−7^ 0.004	175.3
1305	0.086	348.357	8.8452	0.67060	0.11595 0.000	344.788	0.749×10^−7^ 0.004	175.8
1306	0.086	348.045	8.8485	0.61146	0.11607 0.001	344.805	0.737×10^−7^ 0.005	178.7
1307	0.086	347.756	8.8515	0.55506	0.11610 0.001	344.821	0.734×10^−7^ 0.006	179.3
1308	0.086	347.484	8.8544	0.50142	0.11613 0.001	344.838	0.737×10^−7^ 0.006	178.6
1401	0.129	372.502	8.5863	0.77696	0.10982 0.000	368.375	0.701×10^−7^ 0.003	183.7
1402	0.129	372.132	8.5904	0.71422	0.10971 0.000	368.341	0.692×10^−7^ 0.004	185.7
1403	0.129	371.809	8.5940	0.65399	0.10981 0.001	368.273	0.689×10^−7^ 0.004	186.4
1404	0.129	371.503	8.5973	0.59648	0.10998 0.001	368.297	0.694×10^−7^ 0.005	185.3
1405	0.129	371.206	8.6006	0.54161	0.11008 0.001	368.308	0.695×10^−7^ 0.006	185.0
1406	0.129	370.932	8.6036	0.48963	0.11010 0.001	368.298	0.689×10^−7^ 0.006	186.3
1407	0.129	370.634	8.6068	0.43960	0.11006 0.001	368.382	0.685×10^−7^ 0.008	187.4
1408	0.129	370.375	8.6097	0.39278	0.11009 0.001	368.395	0.675×10^−7^ 0.009	190.0
1501	0.391	405.204	8.2181	0.71525	0.10192 0.000	401.334	0.646×10^−7^ 0.004	193.4
1502	0.402	404.847	8.2225	0.65718	0.10181 0.001	401.354	0.632×10^−7^ 0.005	197.2
1503	0.410	404.522	8.2265	0.60151	0.10213 0.000	401.323	0.648×10^−7^ 0.004	192.6
1504	0.415	404.211	8.2302	0.54869	0.10213 0.001	401.382	0.640×10^−7^ 0.006	194.9
1505	0.425	403.927	8.2338	0.49827	0.10236 0.001	401.345	0.650×10^−7^ 0.006	192.2
1506	0.433	403.654	8.2371	0.45015	0.10227 0.001	401.301	0.647×10^−7^ 0.006	192.8
1507	0.439	403.391	8.2403	0.40460	0.10247 0.001	401.345	0.658×10^−7^ 0.007	189.6
1508	0.446	405.536	8.2152	0.77476	0.10197 0.000	401.315	0.651×10^−7^ 0.003	192.0
1601	0.554	426.783	7.9589	0.81341	0.09762 0.000	422.459	0.621×10^−7^ 0.003	199.3
1602	0.558	426.430	7.9634	0.75186	0.09735 0.000	422.522	0.608×10^−7^ 0.003	202.5
1603	0.561	426.075	7.9679	0.69337	0.09752 0.000	422.539	0.616×10^−7^ 0.003	199.9
1604	0.561	425.732	7.9722	0.63755	0.09761 0.000	422.454	0.612×10^−7^ 0.004	201.3
1605	0.562	425.405	7.9763	0.58387	0.09758 0.000	422.551	0.610×10^−7^ 0.004	201.7
1606	0.564	425.096	7.9802	0.53246	0.09763 0.001	422.582	0.606×10^−7^ 0.005	202.9
1607	0.566	424.796	7.9840	0.48350	0.09791 0.001	422.497	0.618×10^−7^ 0.005	199.3
1608	0.569	424.523	7.9875	0.43695	0.09802 0.001	422.474	0.620×10^−7^ 0.006	198.9
1701	0.768	453.898	7.6094	0.86040	0.09206 0.000	449.481	0.584×10^−7^ 0.003	209.1
1702	0.770	453.514	7.6147	0.80056	0.09215 0.000	449.510	0.586×10^−7^ 0.003	208.5
1703	0.772	453.140	7.6199	0.74281	0.09221 0.000	449.496	0.589×10^−7^ 0.003	207.2
1704	0.776	452.793	7.6247	0.68722	0.09222 0.000	449.568	0.586×10^−7^ 0.004	207.9
1705	0.779	452.439	7.6297	0.63370	0.09241 0.000	449.538	0.589×10^−7^ 0.003	207.2
1706	0.781	452.119	7.6341	0.58236	0.09224 0.001	449.544	0.583×10^−7^ 0.005	208.6
1707	0.782	451.799	7.6385	0.53352	0.09243 0.001	449.527	0.586×10^−7^ 0.005	207.6
1708	0.782	451.521	7.6423	0.48659	0.09244 0.001	449.507	0.584×10^−7^ 0.006	208.2
1801	1.023	480.654	7.2293	0.84664	0.08720 0.000	473.650	0.560×10^−7^ 0.003	217.4
1802	1.018	480.279	7.2349	0.78957	0.08709 0.000	473.684	0.561×10^−7^ 0.003	216.6
1803	1.016	479.914	7.2405	0.73476	0.08728 0.000	473.684	0.565×10^−7^ 0.004	215.2
1804	1.014	479.555	7.2459	0.68157	0.08738 0.000	473.713	0.568×10^−7^ 0.003	213.9
1805	1.014	479.212	7.2512	0.63048	0.08739 0.000	473.808	0.568×10^−7^ 0.004	213.7
1806	1.009	478.875	7.2562	0.58183	0.08742 0.001	473.789	0.559×10^−7^ 0.004	217.0
1807	1.008	478.564	7.2609	0.53447	0.08754 0.001	473.801	0.569×10^−7^ 0.005	213.1
1808	1.008	478.262	7.2655	0.48937	0.08764 0.001	473.753	0.564×10^−7^ 0.005	215.3
1901	1.681	504.349	6.8718	0.88755	0.08337 0.000	497.373	0.554×10^−7^ 0.003	221.5
1902	1.673	503.948	6.8784	0.82922	0.08338 0.000	497.373	0.560×10^−7^ 0.003	218.8
1903	1.669	503.572	6.8847	0.77381	0.08352 0.000	497.404	0.566×10^−7^ 0.003	216.3
1904	1.662	503.194	6.8909	0.71986	0.08347 0.000	497.340	0.565×10^−7^ 0.003	216.4
1905	1.659	502.816	6.8973	0.66827	0.08356 0.000	497.278	0.551×10^−7^ 0.003	221.6
1906	1.654	502.464	6.9031	0.61856	0.08366 0.000	497.298	0.554×10^−7^ 0.004	220.4
1907	1.651	502.127	6.9088	0.57068	0.08375 0.001	497.277	0.553×10^−7^ 0.004	220.8
1908	1.647	501.825	6.9138	0.52450	0.08380 0.001	497.309	0.558×10^−7^ 0.005	218.5
2001	2.293	526.378	6.4964	0.87427	0.08046 0.000	519.727	0.542×10^−7^ 0.003	230.8
2002	2.295	525.971	6.5049	0.81718	0.08046 0.000	519.611	0.542×10^−7^ 0.003	230.5
2003	2.297	525.577	6.5132	0.76206	0.08063 0.000	519.568	0.549×10^−7^ 0.003	227.8
2004	2.299	525.193	6.5212	0.70904	0.08059 0.000	519.685	0.547×10^−7^ 0.004	228.1
2005	2.301	524.830	6.5287	0.65793	0.08079 0.000	519.738	0.547×10^−7^ 0.004	228.2
2006	2.302	524.479	6.5359	0.60865	0.08078 0.000	519.685	0.545×10^−7^ 0.004	228.5
2007	2.305	524.144	6.5429	0.56137	0.08089 0.001	519.685	0.550×10^−7^ 0.004	226.4
2008	2.306	523.830	6.5493	0.51601	0.08103 0.001	519.706	0.558×10^−7^ 0.006	223.1
2101	2.682	554.337	5.8477	0.86413	0.07653 0.001	548.030	0.516×10^−7^ 0.007	254.8
2102	2.683	553.911	5.8611	0.80784	0.07659 0.001	548.130	0.517×10^−7^ 0.007	253.6
2103	2.684	553.515	5.8736	0.75366	0.07672 0.001	548.063	0.523×10^−7^ 0.008	250.8
2104	2.686	553.111	5.8862	0.70126	0.07680 0.001	548.109	0.524×10^−7^ 0.006	250.2
2105	2.686	552.722	5.8981	0.65069	0.07686 0.001	548.140	0.522×10^−7^ 0.009	250.7
2106	2.688	552.367	5.9089	0.60212	0.07703 0.001	548.129	0.533×10^−7^ 0.009	245.6
2107	2.688	552.041	5.9187	0.55544	0.07702 0.001	548.132	0.532×10^−7^ 0.010	245.6
2108	2.691	551.695	5.9294	0.51041	0.07709 0.001	548.141	0.534×10^−7^ 0.011	244.1
2109	19.346	553.364	6.9566	0.86455	0.09108 0.000	548.247	0.632×10^−7^ 0.003	211.7
2110	19.353	552.979	6.9613	0.80814	0.09119 0.000	548.194	0.633×10^−7^ 0.004	211.2
2111	19.357	552.629	6.9654	0.75383	0.09120 0.000	548.217	0.631×10^−7^ 0.004	211.5
2112	19.358	552.276	6.9695	0.70146	0.09107 0.001	548.226	0.619×10^−7^ 0.004	214.9
2113	14.335	553.521	6.7600	0.86425	0.08774 0.000	548.096	0.607×10^−7^ 0.003	218.3
2114	14.335	553.155	6.7648	0.80806	0.08775 0.000	548.075	0.608×10^−7^ 0.003	217.4
2115	14.335	552.794	6.7695	0.75378	0.08789 0.001	548.132	0.613×10^−7^ 0.004	215.5
2116	14.336	552.424	6.7744	0.70141	0.08778 0.001	548.153	0.603×10^−7^ 0.005	218.6
2117	9.599	553.745	6.5167	0.86414	0.08408 0.000	548.099	0.586×10^−7^ 0.004	224.4
2118	9.599	553.363	6.5227	0.80789	0.08418 0.000	548.088	0.592×10^−7^ 0.004	221.9
2119	9.599	553.003	6.5283	0.75370	0.08423 0.001	548.045	0.598×10^−7^ 0.004	219.2
2120	9.598	552.630	6.5341	0.70139	0.08420 0.001	548.065	0.592×10^−7^ 0.004	221.1
2121	6.941	553.927	6.3343	0.86406	0.08179 0.000	548.065	0.579×10^−7^ 0.004	226.7
2122	6.941	553.531	6.3415	0.80788	0.08192 0.000	548.078	0.587×10^−7^ 0.004	223.6
2123	6.941	553.133	6.3487	0.75367	0.08202 0.001	548.045	0.589×10^−7^ 0.005	222.4
2124	6.942	552.777	6.3552	0.70128	0.08199 0.001	548.065	0.588×10^−7^ 0.005	222.5
2125	4.512	554.119	6.1081	0.86378	0.07885 0.000	548.033	0.550×10^−7^ 0.004	237.6
2126	4.512	553.711	6.1173	0.80730	0.07890 0.001	548.023	0.556×10^−7^ 0.004	234.7
2127	4.512	553.323	6.1261	0.75307	0.07904 0.000	548.033	0.567×10^−7^ 0.004	230.2
2128	4.512	552.936	6.1348	0.70093	0.07910 0.001	548.034	0.565×10^−7^ 0.004	230.4

**Table 2:** Thermal conductivity, thermal diffusivity, and heat capacity of argon gas near 300 K

Run Pt.	Pressure MPa	Temperature K	Density mol/L	Power W/m	Thermal conductivity W/(m·K) STAT	Cell temperature K	Thermal diffusivity m^2^/s DSTAT	Specific heat J/(mol·K)
1001	65.224	301.925	19.9271	0.33737	0.05378 0.000	298.178	0.795×10^−7^ 0.004	33.9
1002	65.224	301.453	19.9550	0.29703	0.05381 0.000	298.145	0.772×10^−7^ 0.004	34.9
1003	65.224	301.021	19.9805	0.25949	0.05392 0.001	298.188	0.768×10^−7^ 0.005	35.1
1004	65.223	300.621	20.0041	0.22432	0.05404 0.001	298.152	0.767×10^−7^ 0.006	35.2
1005	60.534	301.982	19.1757	0.33733	0.05128 0.000	298.079	0.772×10^−7^ 0.004	34.6
1006	60.533	301.554	19.2011	0.29699	0.05131 0.001	298.069	0.774×10^−7^ 0.005	34.5
1007	60.533	301.101	19.2281	0.25926	0.05136 0.001	298.067	0.771×10^−7^ 0.005	34.6
1008	60.531	300.698	19.2520	0.22406	0.05141 0.001	298.105	0.768×10^−7^ 0.006	34.7
1009	56.254	301.663	18.4516	0.29684	0.04899 0.000	298.073	0.753×10^−7^ 0.004	35.1
1010	56.254	301.195	18.4801	0.25917	0.04905 0.001	298.029	0.758×10^−7^ 0.005	34.9
1011	56.254	300.770	18.5058	0.22406	0.04907 0.001	298.081	0.753×10^−7^ 0.006	35.1
1012	56.256	300.350	18.5315	0.19174	0.04925 0.001	298.081	0.757×10^−7^ 0.007	35.1
1013	52.289	301.803	17.6955	0.29714	0.04683 0.001	298.017	0.750×10^−7^ 0.004	35.2
1014	52.289	301.333	17.7241	0.25931	0.04687 0.001	298.049	0.755×10^−7^ 0.005	34.9
1015	52.289	300.877	17.7519	0.22425	0.04696 0.001	298.047	0.750×10^−7^ 0.006	35.2
1016	52.288	300.462	17.7771	0.19162	0.04702 0.001	298.051	0.746×10^−7^ 0.007	35.4
1017	48.788	301.940	16.9726	0.29698	0.04486 0.000	298.026	0.749×10^−7^ 0.004	35.1
1018	48.789	301.451	17.0025	0.25927	0.04492 0.001	298.000	0.752×10^−7^ 0.004	35.0
1019	48.789	300.978	17.0314	0.22414	0.04496 0.001	298.019	0.749×10^−7^ 0.005	35.1
1020	48.788	300.553	17.0573	0.19154	0.04504 0.001	298.031	0.751×10^−7^ 0.007	35.0
1021	45.435	302.110	16.2241	0.29672	0.04306 0.000	298.010	0.769×10^−7^ 0.003	34.3
1022	45.435	301.595	16.2553	0.25911	0.04304 0.001	297.992	0.763×10^−7^ 0.004	34.5
1023	45.435	301.120	16.2842	0.22393	0.04302 0.001	298.020	0.762×10^−7^ 0.005	34.5
1024	45.435	300.652	16.3128	0.19141	0.04308 0.001	297.990	0.765×10^−7^ 0.006	34.4
1025	42.251	301.754	15.4902	0.25937	0.04117 0.001	298.003	0.749×10^−7^ 0.005	35.2
1026	42.251	301.239	15.5212	0.22413	0.04119 0.001	297.997	0.742×10^−7^ 0.005	35.5
1027	42.249	300.762	15.5498	0.19151	0.04127 0.001	298.024	0.736×10^−7^ 0.006	35.9
1028	42.249	300.317	15.5767	0.16149	0.04131 0.001	297.949	0.727×10^−7^ 0.008	36.4
1029	39.526	301.865	14.7903	0.25936	0.03955 0.000	298.007	0.749×10^−7^ 0.004	35.4
1030	39.526	301.334	14.8219	0.22432	0.03959 0.001	297.965	0.751×10^−7^ 0.005	35.3
1031	39.526	300.853	14.8508	0.19179	0.03965 0.001	297.975	0.751×10^−7^ 0.006	35.3
1032	39.525	300.389	14.8785	0.16183	0.03975 0.001	298.012	0.748×10^−7^ 0.008	35.5
1033	36.708	302.037	14.0145	0.25981	0.03800 0.000	298.005	0.774×10^−7^ 0.004	34.7
1034	36.708	301.493	14.0464	0.22461	0.03791 0.001	298.010	0.762×10^−7^ 0.005	35.1
1035	36.710	300.960	14.0781	0.19185	0.03794 0.001	298.041	0.759×10^−7^ 0.005	35.3
1036	36.710	300.488	14.1059	0.16175	0.03798 0.001	298.019	0.756×10^−7^ 0.007	35.4
1037	33.968	302.077	13.2166	0.25240	0.03622 0.001	297.990	0.773×10^−7^ 0.005	35.0
1038	33.968	301.609	13.2432	0.22419	0.03627 0.001	297.950	0.777×10^−7^ 0.005	34.9
1039	33.969	301.175	13.2683	0.19786	0.03626 0.001	297.929	0.779×10^−7^ 0.005	34.8
1040	33.969	300.784	13.2907	0.17318	0.03627 0.001	297.930	0.773×10^−7^ 0.007	35.1
1041	31.748	302.007	12.5367	0.23797	0.03491 0.000	297.938	0.806×10^−7^ 0.004	34.1
1042	31.748	301.535	12.5629	0.21097	0.03489 0.001	297.946	0.795×10^−7^ 0.005	34.6
1043	31.748	301.096	12.5873	0.18554	0.03492 0.001	297.926	0.795×10^−7^ 0.006	34.6
1044	31.749	300.678	12.6109	0.16143	0.03497 0.001	297.941	0.796×10^−7^ 0.007	34.5
1045	29.280	301.947	11.7377	0.22396	0.03347 0.000	297.936	0.839×10^−7^ 0.004	33.6
1046	29.281	301.476	11.7630	0.19797	0.03353 0.001	297.935	0.849×10^−7^ 0.005	33.2
1047	29.281	301.044	11.7859	0.17332	0.03355 0.001	297.909	0.840×10^−7^ 0.006	33.5
1048	29.282	300.612	11.8092	0.15021	0.03347 0.001	297.956	0.826×10^−7^ 0.007	34.0
1049	27.318	301.897	11.0699	0.21088	0.03216 0.000	297.971	0.836×10^−7^ 0.004	34.3
1050	27.319	301.427	11.0940	0.18535	0.03212 0.001	297.945	0.830×10^−7^ 0.005	34.5
10S1	27.319	300.963	11.1178	0.16128	0.03217 0.001	297.930	0.832×10^−7^ 0.006	34.4
1052	27.320	300.558	11.1389	0.13912	0.03225 0.001	297.957	0.836×10^−7^ 0.008	34.3
1053	25.276	302.085	10.3335	0.21027	0.03091 0.001	297.951	0.890×10^−7^ 0.004	33.1
1054	25.276	301.603	10.3566	0.18491	0.03092 0.001	297.979	0.884×10^−7^ 0.005	33.3
1055	25.276	301.123	10.3796	0.16112	0.03089 0.001	297.932	0.879×10^−7^ 0.006	33.4
1056	25.276	300.690	10.4005	0.13915	0.03096 0.001	297.956	0.881×10^−7^ 0.007	33.4
1057	23.207	302.038	9.5687	0.19758	0.02966 0.000	298.009	0.928×10^−7^ 0.004	32.8
1058	23.207	301.529	9.5916	0.17297	0.02969 0.001	297.980	0.936×10^−7^ 0.005	32.6
1059	23.208	301.072	9.6124	0.14995	0.02968 0.001	298.006	0.933×10^−7^ 0.006	32.7
1060	23.208	300.634	9.6321	0.12854	0.02967 0.001	298.007	0.926×10^−7^ 0.008	32.9
1061	21.499	301.961	8.9175	0.18527	0.02868 0.001	297.969	0.100×10^−6^ 0.005	31.4
1062	21.499	301.451	8.9387	0.16130	0.02862 0.001	297.983	0.991×10^−7^ 0.006	31.8
1063	21.499	301.206	8.9490	0.14993	0.02864 0.001	297.953	0.998×10^−7^ 0.006	31.6
1064	21.499	300.536	8.9771	0.11843	0.02867 0.001	297.978	0.100×10^−6^ 0.009	31.5
1065	19.660	301.868	8.1962	0.17302	0.02753 0.001	297.975	0.101×10^−6^ 0.005	32.6
1066	19.660	301.342	8.2163	0.14986	0.02753 0.001	297.986	0.100×10^−6^ 0.006	32.9
1067	19.660	300.856	8.2350	0.12845	0.02753 0.001	297.981	0.989×10^−7^ 0.008	33.4
1068	19.662	300.399	8.2532	0.10874	0.02756 0.001	298.003	0.976×10^−7^ 0.009	33.8
1069	17.864	302.035	7.4644	0.17310	0.02643 0.001	298.005	0.111×10^−6^ 0.006	31.4
1070	17.864	301.489	7.4833	0.15007	0.02642 0.001	297.966	0.109×10^−6^ 0.006	31.7
1071	17.864	300.981	7.5010	0.12874	0.02644 0.001	297.933	0.109×10^−6^ 0.007	32.0
1072	17.864	300.516	7.5173	0.10900	0.02647 0.001	297.960	0.108×10^−6^ 0.010	32.3
1073	16.141	302.241	6.7481	0.17324	0.02545 0.001	297.971	0.121×10^−6^ 0.005	30.5
1074	16.141	301.664	6.7659	0.15023	0.02542 0.001	297.956	0.119×10^−6^ 0.006	30.9
1075	16.141	301.119	6.7828	0.12876	0.02544 0.001	297.989	0.120×10^−6^ 0.007	30.8
1076	16.141	300.607	6.7988	0.10905	0.02546 0.001	297.956	0.119×10^−6^ 0.009	31.0
1077	14.442	302.139	6.0414	0.16149	0.02446 0.001	297.970	0.133×10^−6^ 0.005	29.7
1078	14.442	301.576	6.0568	0.13912	0.02445 0.001	297.955	0.133×10^−6^ 0.006	29.7
1079	14.442	301.049	6.0712	0.11858	0.02442 0.001	297.977	0.132×10^−6^ 0.007	30.1
1080	14.442	300.563	6.0846	0.09963	0.02446 0.001	297.960	0.132×10^−6^ 0.010	30.1
1081	12.754	302.079	5.3298	0.14979	0.02351 0.001	297.927	0.151×10^−6^ 0.006	28.6
1082	12.754	301.496	5.3436	0.12843	0.02348 0.001	297.950	0.149×10^−6^ 0.006	29.0
1083	12.754	300.934	5.3569	0.10882	0.02352 0.001	298.002	0.149×10^−6^ 0.008	29.0
1084	12.754	300.446	5.3685	0.09075	0.02349 0.001	298.003	0.148×10^−6^ 0.011	29.2
1085	10.898	302.004	4.5421	0.13917	0.02253 0.001	297.935	0.172×10^−6^ 0.006	28.1
1086	10.898	301.417	4.5539	0.11851	0.02251 0.001	297.945	0.171×10^−6^ 0.007	28.4
1087	10.898	300.864	4.5648	0.09964	0.02251 0.001	297.996	0.169×10^−6^ 0.009	28.7
1088	10.898	300.355	4.5748	0.08236	0.02251 0.001	297.951	0.166×10^−6^ 0.012	29.3
1089	9.295	302.244	3.8552	0.13887	0.02171 0.001	297.960	0.205×10^−6^ 0.006	26.8
1090	9.295	301.611	3.8654	0.11841	0.02167 0.001	297.949	0.202×10^−6^ 0.007	27.2
1091	9.295	301.033	3.8748	0.09956	0.02166 0.001	297.938	0.200×10^−6^ 0.009	27.5
1092	9.294	300.501	3.8832	0.08235	0.02169 0.001	297.979	0.199×10^−6^ 0.012	27.7
1093	7.510	302.191	3.0984	0.12862	0.02085 0.001	297.991	0.247×10^−6^ 0.006	26.5
1093	7.510	301.544	3.1064	0.10882	0.02082 0.001	297.941	0.243×10^−6^ 0.008	27.0
1095	7.510	300.939	3.1138	0.09061	0.02083 0.001	297.995	0.245×10^−6^ 0.010	26.8
1096	7.509	300.403	3.1203	0.07423	0.02084 0.001	297.975	0.244×10^−6^ 0.014	27.0
1097	5.829	302.136	2.3897	0.11818	0.02008 0.001	297.999	0.324×10^−6^ 0.007	25.2
1098	5.828	301.479	2.3955	0.09942	0.02008 0.001	297.970	0.324×10^−6^ 0.009	25.3
1099	5.827	300.879	2.4009	0.08221	0.02004 0.001	297.970	0.319×10^−6^ 0.012	25.7
1100	5.827	300.298	2.4060	0.06671	0.02009 0.002	297.950	0.307×10^−6^ 0.015	26.8
1101	4.375	302.061	1.7826	0.10864	0.01960 0.001	297.985	0.429×10^−6^ 0.008	25.0
1102	4.375	301.373	1.7872	0.09059	0.01952 0.001	297.980	0.413×10^−6^ 0.011	25.9
1103	4.375	300.743	1.7915	0.07418	0.01951 0.001	297.997	0.403×10^−6^ 0.014	26.5
1104	4.375	300.195	1.7952	0.05939	0.01948 0.002	298.010	0.388×10^−6^ 0.019	27.6
1105	2.601	302.360	1.0495	0.10871	0.01893 0.001	297.970	0.693×10^−6^ 0.010	25.3
1106	2.601	301.634	1.0522	0.09071	0.01887 0.001	297.950	0.676×10^−6^ 0.011	25.9
1107	2.600	300.980	1.0544	0.07435	0.01886 0.001	297.999	0.655×10^−6^ 0.014	26.8
1108	2.600	300.356	1.0567	0.05952	0.01885 0.002	297.976	0.631×10^−6^ 0.018	27.9
1109	65.509	302.887	19.9141	0.41761	0.05374 0.000	298.353	0.761×10^−7^ 0.004	35.4
1110	65.499	302.474	19.9368	0.38071	0.05378 0.000	298.313	0.759×10^−7^ 0.004	35.4
1111	65.484	302.092	19.9570	0.34638	0.05388 0.000	298.261	0.778×10^−7^ 0.004	34.7
1112	65.474	301.728	19.9769	0.31306	0.05387 0.001	298.282	0.778×10^−7^ 0.004	34.6
1113	65.468	301.382	19.9964	0.28165	0.05389 0.001	298.266	0.780×10^−7^ 0.005	34.5
1114	65.454	301.034	20.0147	0.25238	0.05396 0.001	298.257	0.765×10^−7^ 0.006	35.2
1115	65.447	300.704	20.0331	0.22404	0.05408 0.001	298.216	0.767×10^−7^ 0.007	35.1
1116	65.437	300.433	20.0478	0.19763	0.05405 0.001	298.226	0.757×10^−7^ 0.008	35.6
1117	65.430	300.168	20.0624	0.17284	0.05403 0.001	298.269	0.780×10^−7^ 0.009	34.5
1118	65.422	299.903	20.0769	0.14987	0.05407 0.001	298.303	0.764×10^−7^ 0.011	35.2
1119	65.414	299.654	20.0905	0.12847	0.05410 0.002	298.231	0.748×10^−7^ 0.014	36.0
1120	65.407	299.444	20.1018	0.10873	0.05413 0.002	298.267	0.733×10^−7^ 0.017	36.7
1121	65.353	299.421	20.0951	0.10852	0.05406 0.002	298.212	0.756×10^−7^ 0.016	35.6
1122	65.348	299.641	20.0812	0.12835	0.05405 0.002	298.269	0.773×10^−7^ 0.014	34.8
1123	65.338	299.869	20.0662	0.14973	0.05401 0.001	298.227	0.766×10^−7^ 0.011	35.1
1124	65.331	300.121	20.0501	0.17277	0.05405 0.001	298.222	0.783×10^−7^ 0.009	34.4
1125	65.322	300.404	20.0319	0.19739	0.05395 0.001	298.222	0.780×10^−7^ 0.008	34.5
1126	65.314	300.682	20.0143	0.22362	0.05389 0.001	298.219	0.776×10^−7^ 0.007	34.7
1127	65.306	301.000	19.9942	0.25163	0.05376 0.001	298.224	0.770×10^−7^ 0.006	34.9
1128	65.299	301.327	19.9738	0.28120	0.05370 0.001	298.217	0.770×10^−7^ 0.005	34.9
1129	65.290	301.675	19.9519	0.31240	0.05367 0.001	298.244	0.770×10^−7^ 0.005	34.9
1130	65.282	302.034	19.9296	0.34525	0.05364 0.000	298.232	0.771×10^−7^ 0.004	34.8
1131	65.273	302.431	19.9049	0.37948	0.05352 0.001	298.212	0.767×10^−7^ 0.005	35.0
1132	65.265	302.841	19.8796	0.41638	0.05343 0.000	298.206	0.762×10^−7^ 0.004	35.2
1133	65.220	302.836	19.8731	0.41590	0.05346 0.000	298.170	0.775×10^−7^ 0.004	34.6
1134	65.213	302.439	19.8953	0.37977	0.05347 0.000	298.182	0.776×10^−7^ 0.004	34.6
1135	65.207	302.037	19.9180	0.34562	0.05357 0.000	298.201	0.777×10^−7^ 0.004	34.5
1136	65.200	301.673	19.9383	0.31300	0.05366 0.001	298.244	0.776×10^−7^ 0.005	34.6
1137	65.191	301.313	19.9583	0.28163	0.05372 0.001	298.227	0.777×10^−7^ 0.005	34.6
1138	65.185	300.992	19.9762	0.25205	0.05372 0.001	298.244	0.772×10^−7^ 0.006	34.8
1139	65.176	300.680	19.9934	0.22382	0.05377 0.001	298.224	0.771×10^−7^ 0.007	34.8
1140	65.170	300.369	20.0108	0.19759	0.05385 0.001	298.162	0.769×10^−7^ 0.008	35.0
1141	65.164	300.101	20.0259	0.17300	0.05388 0.001	298.207	0.765×10^−7^ 0.009	35.1
1142	65.159	299.841	20.0405	0.14996	0.05389 0.001	298.195	0.760×10^−7^ 0.011	35.3
1143	65.154	299.607	20.0537	0.12861	0.05404 0.002	298.214	0.760×10^−7^ 0.014	35.5
1144	65.146	299.385	20.0658	0.10884	0.05402 0.002	298.173	0.733×10^−7^ 0.017	36.7

**Table 3:** Thermal conductivity, thermal diffusivity, and heat capacity of nitrogen gas near 425 K

Run Pt.	Pressure MPa	Temperature K	Density mol/L	Power W/m	Thermal conductivity W/(m·K) STAT	Cell temperature K	Thermal diffusivity m^2^/s DSTAT	Specific heat J/(mol·K)
4001	67.472	426.243	13.0983	0.41718	0.06062 0.001	421.862	0.122×10^−6^ 0.008	37.8
4002	67.472	425.770	13.1098	0.37241	0.06062 0.001	421.855	0.121×10^−6^ 0.009	38.1
4003	67.471	425.332	13.1205	0.33045	0.06063 0.001	421.844	0.122×10^−6^ 0.011	37.8
4004	67.470	424.918	13.1305	0.29096	0.06059 0.002	421.887	0.120×10^−6^ 0.014	38.1
4005	58.114	426.577	11.9010	0.41738	0.05671 0.001	421.881	0.125×10^−6^ 0.008	37.8
4006	58.113	426.072	11.9128	0.37279	0.05672 0.001	421.854	0.125×10^−6^ 0.010	37.8
4007	58.113	425.597	11.9239	0.33059	0.05668 0.001	421.851	0.124×10^−6^ 0.011	38.1
4008	58.112	425.159	11.9341	0.29111	0.05670 0.001	421.932	0.124×10^−6^ 0.013	38.0
4009	53.330	426.824	11.2302	0.41741	0.05472 0.001	421.932	0.129×10^−6^ 0.009	37.5
4010	53.331	426.299	11.2424	0.37276	0.05475 0.001	421.896	0.129×10^−6^ 0.008	37.5
4011	53.331	425.804	11.2537	0.33063	0.05473 0.001	421.910	0.128×10^−6^ 0.011	37.6
4012	53.330	425.351	11.2640	0.29114	0.05469 0.001	421.893	0.128×10^−6^ 0.012	37.8
4013	48.692	427.059	10.5387	0.41713	0.05282 0.001	421.976	0.134×10^−6^ 0.009	36.9
4014	48.692	426.504	10.5510	0.37264	0.05285 0.001	421.986	0.134×10^−6^ 0.009	37.0
4015	48.694	426.006	10.5622	0.33066	0.05278 0.001	421.980	0.133×10^−6^ 0.010	37.2
4016	48.695	425.535	10.5728	0.29113	0.05275 0.001	421.999	0.132×10^−6^ 0.012	37.4
4017	43.950	427.314	9.7851	0.41691	0.05090 0.001	421.968	0.141×10^−6^ 0.008	36.3
4018	43.950	426.757	9.7970	0.37232	0.05088 0.001	422.007	0.141×10^−6^ 0.008	36.5
4019	43.951	426.234	9.8081	0.33020	0.05082 0.001	421.962	0.140×10^−6^ 0.010	36.5
4020	43.952	425.736	9.8188	0.29078	0.05084 0.001	421.988	0.142×10^−6^ 0.012	36.2
4021	39.833	427.590	9.0889	0.41683	0.04918 0.001	422.036	0.150×10^−6^ 0.008	35.6
4022	39.833	426.999	9.1006	0.37243	0.04916 0.001	422.048	0.149×10^−6^ 0.009	35.7
4023	39.833	426.441	9.1120	0.33052	0.04915 0.001	422.045	0.149×10^−6^ 0.010	35.8
4024	39.833	425.924	9.1224	0.29100	0.04915 0.001	422.039	0.150×10^−6^ 0.012	35.6
4025	35.790	427.900	8.3645	0.41754	0.04757 0.001	422.152	0.160×10^−6^ 0.008	34.9
4026	35.790	427.305	8.3758	0.37311	0.04752 0.001	422.153	0.160×10^−6^ 0.008	35.0
4027	35.790	426.735	8.3864	0.33098	0.04749 0.001	422.110	0.160×10^−6^ 0.010	35.0
4028	35.790	426.179	8.3970	0.29138	0.04746 0.001	422.150	0.158×10^−6^ 0.012	35.3
4029	32.279	427.535	7.7127	0.37281	0.04613 0.001	422.149	0.171×10^−6^ 0.009	34.5
4030	32.278	426.922	7.7235	0.33073	0.04609 0.001	422.178	0.169×10^−6^ 0.010	34.7
4031	32.277	426.336	7.7338	0.29120	0.04609 0.001	422.166	0.166×10^−6^ 0.012	35.5
4032	32.277	425.813	7.7430	0.25423	0.04601 0.001	422.149	0.163×10^−6^ 0.013	36.0
4033	28.758	427.433	7.0199	0.37227	0.04475 0.001	421.825	0.182×10^−6^ 0.009	34.5
4034	28.758	426.811	7.0301	0.33032	0.04468 0.001	421.816	0.179×10^−6^ 0.010	34.9
4035	28.758	426.211	7.0400	0.29088	0.04468 0.001	421.790	0.179×10^−6^ 0.011	35.0
4036	28.758	425.639	7.0495	0.25398	0.04463 0.001	421.822	0.176×10^−6^ 0.013	35.6
4037	25.269	427.643	6.2935	0.37238	0.04336 0.001	421.789	0.200×10^−6^ 0.009	33.9
4038	25.269	426.975	6.3034	0.33045	0.04330 0.001	421.785	0.197×10^−6^ 0.010	34.3
4039	25.269	426.356	6.3127	0.29108	0.04329 0.001	421.807	0.196×10^−6^ 0.011	34.5
4040	25.268	425.804	6.3208	0.25421	0.04325 0.001	421.784	0.195×10^−6^ 0.013	34.6
4041	22.030	427.617	5.5903	0.35533	0.04207 0.001	421.789	0.222×10^−6^ 0.009	33.3
4042	22.029	427.074	5.5975	0.32252	0.04206 0.001	421.800	0.222×10^−6^ 0.010	33.3
4043	22.029	426.577	5.6040	0.29091	0.04201 0.001	421.786	0.221×10^−6^ 0.011	33.5
4044	22.028	426.084	5.6105	0.26121	0.04198 0.001	421.793	0.218×10^−6^ 0.013	33.8
4045	19.120	427.585	4.9318	0.33856	0.04095 0.001	421.825	0.246×10^−6^ 0.010	33.0
4046	19.120	427.026	4.9385	0.30638	0.04090 0.001	421.830	0.246×10^−6^ 0.011	33.0
4047	19.120	426.514	4.9446	0.27586	0.04086 0.001	421.829	0.244×10^−6^ 0.012	33.3
4048	19.120	426.029	4.9504	0.24689	0.04086 0.001	421.844	0.245×10^−6^ 0.013	33.3
4049	16.150	427.870	4.2299	0.33858	0.03985 0.001	421.893	0.281×10^−6^ 0.009	32.8
4050	16.150	427.308	4.2357	0.30642	0.03982 0.001	421.888	0.279×10^−6^ 0.011	33.1
4051	16.150	426.767	4.2413	0.27587	0.03982 0.001	421.892	0.280×10^−6^ 0.012	33.0
4052	16.150	426.256	4.2466	0.24689	0.03979 0.001	421.882	0.277×10^−6^ 0.013	33.3
4053	13.434	427.860	3.5681	0.32224	0.03885 0.001	421.902	0.332×10^−6^ 0.011	32.1
4054	13.433	427.263	3.5731	0.29090	0.03880 0.001	421.891	0.329×10^−6^ 0.012	32.4
4055	13.433	426.739	3.5777	0.26115	0.03879 0.001	421.927	0.328×10^−6^ 0.012	32.4
4056	13.433	426.225	3.5820	0.23294	0.03873 0.001	421.889	0.326×10^−6^ 0.014	32.7
4057	10.616	428.169	2.8565	0.32218	0.03788 0.001	421.961	0.404×10^−6^ 0.011	32.0
4058	10.615	427.574	2.8603	0.29100	0.03784 0.001	421.930	0.403×10^−6^ 0.012	32.1
4059	10.615	427.001	2.8643	0.26120	0.03782 0.001	421.955	0.405×10^−6^ 0.014	31.9
4060	10.614	426.476	2.8678	0.23298	0.03782 0.001	421.964	0.407×10^−6^ 0.015	31.8
4061	8.185	428.204	2.2268	0.30620	0.03705 0.001	421.986	0.511×10^−6^ 0.012	31.8
4062	8.184	427.588	2.2299	0.27571	0.03701 0.001	421.956	0.506×10^−6^ 0.012	32.1
4063	8.184	427.013	2.2329	0.24678	0.03700 0.001	421.992	0.509×10^−6^ 0.014	31.9
4064	8.184	426.459	2.2359	0.21939	0.03696 0.002	421.986	0.504×10^−6^ 0.016	32.2
4065	5.684	428.304	1.5628	0.29076	0.03627 0.001	422.071	0.732×10^−6^ 0.012	30.9
4066	5.684	427.662	1.5650	0.26101	0.03625 0.001	422.046	0.733×10^−6^ 0.014	30.9
4067	5.683	427.047	1.5671	0.23291	0.03624 0.001	421.984	0.714×10^−6^ 0.016	31.8
4068	5.682	426.467	1.5692	0.20636	0.03621 0.002	422.052	0.714×10^−6^ 0.017	31.7
4069	3.262	428.449	0.9050	0.27577	0.03563 0.001	422.058	0.122×10^−6^ 0.015	31.5
4070	3.260	427.778	0.9061	0.24681	0.03562 0.001	422.127	0.122×10^−6^ 0.016	31.5
4071	3.259	427.155	0.9071	0.21944	0.03561 0.002	422.067	0.121×10^−6^ 0.018	31.8
4072	3.258	426.571	0.9082	0.19367	0.03557 0.002	422.071	0.122×10^−6^ 0.020	31.6

## Footnotes

2 This is possible because, as shown later, *Q*_1_/L*q*≃2 × 10^−2^ for the transient hot-wire instrument and, therefore, an error of 0.1% in *Q*_1_produces an error of 0.002% in *q*, well beyond the experimental accuracy.
